# Reduced Level of the BCL11B Protein Is Associated with Adult T-Cell Leukemia/Lymphoma

**DOI:** 10.1371/journal.pone.0055147

**Published:** 2013-01-30

**Authors:** Nobuyuki Kurosawa, Rika Fujimoto, Tatsuhiko Ozawa, Takahiro Itoyama, Naoki Sadamori, Masaharu Isobe

**Affiliations:** 1 Faculty of Science and Engineering, Graduate School, University of Toyama, Toyama, Japan; 2 Department of Immunology, Kochi Medical School, Kochi, Japan; 3 Department of Immunology, Graduate School of Medicine and Pharmaceutical Sciences for Research, University of Toyama, Toyama, Japan; 4 Department of Hematology, Imamura Bun-in Hospital, Kagoshima, Japan; 5 Department of Nursing, Faculty of Nursing and Nutrition, University of Nagasaki, Nagasaki, Japan; University of Navarra, Center for Applied Medical Research, Spain

## Abstract

**Background:**

Adult T-cell leukemia/lymphoma (ATLL) develops in a small proportion of human T-cell leukemia virus type I (HTLV-I)-infected individuals. However, the mechanism by which HTLV-I causes ATLL has not been fully elucidated. To provide fundamental insights into the multistep process of leukemogenesis, we have mapped the chromosomal abnormalities in 50 ATLL cases to identify potential key regulators of ATLL.

**Results:**

The analysis of breakpoints in one ATLL case with the translocations t(14;17)(q32;q22-23) resulted in the identification of a Kruppel zinc finger gene, *BCL11B*, which plays a crucial role in T-cell development. Among the 7 ATLL cases that we examined by immunofluorescence analysis, 4 displayed low and one displayed moderate BCL11B signal intensities. A dramatically reduced level of the BCL11B protein was also found in HTLV-I-positive T-cell lines. The ectopic expression of *BCL11B* resulted in significant growth suppression in ATLL-derived cell lines but not in Jurkat cells.

**Conclusions:**

Our genetic and functional data provide the first evidence that a reduction in the level of the BCL11B protein is a key event in the multistep progression of ATLL leukemogenesis.

## Introduction

Adult T-cell leukemia/lymphoma (ATLL) is an aggressive malignant disease of mature CD4+ regulatory T lymphocytes [1]. Human T-cell lymphotropic virus type I (HTLV-I) causes ATLL in a small percentage of infected individuals after a long latency period of multiple years [2]. Several lines of evidence have established that the viral oncoprotein Tax plays a central role, at least during the early stages of leukemogenesis [3]. However, freshly isolated ATLL cells from patients frequently lose Tax protein expression via several mechanisms, resulting in the loss of its pleiotropic effects. Recently, the *HTLV-I bZIP* gene was shown to be consistently expressed in ATLL cells, suggesting that it might play a functional role in cellular transformation and leukemogenesis [4]. Alternatively, based on the long clinical latency of HTLV-I and the low percentage of infected individuals who develop ATLL, the progression to ATLL is believed to be the result of a series of cellular alterations [5], [6]. Thus, the proteins or genes that are specifically altered in ATLL cells are good candidates to evaluate their potential involvement in leukemogenesis. Recently, the profiling of microRNA signatures of ATLL has revealed the activation of NF-kB through the genetic and epigenetic loss of *miR-31* [7].

Karyotypes of ATLL cells exhibit a high degree of diversity and complexity, and recurrent chromosomal abnormalities are rarely observed. However, a precise analysis has demonstrated that breakpoints tend to cluster at specific chromosomal regions, including 1p22, 1q10-21, 2q31-34, 3q, 3q10-12, 3q21, 14q32, and 17q [8]. Recent multicolor spectral karyotyping analysis has revealed frequent chromosomal breakpoints in 10p11, 14q11 and 14q32, and *TCF8* was identified as a candidate tumor suppressor gene within the breakpoint cluster regions in 10p11.2 [9]. The chromosome 14q32 is involved in various types of lymphoid malignancies and harbors several candidate genes that might confer the specific biological aspects of ATLL pathogenesis, such as *TLC1*, *TCL6*, *TML* and *BCL11B* [10]–[14]. *BCL11B* functions as a transcriptional regulator by directly or indirectly binding to specific DNA sequences and recruiting co-repressor complexes [15]–[18]. *BCL11B* plays a crucial role in T-cell development and has been implicated in human T-cell acute lymphoblastic leukemia [19]–[23]. The region on mouse chromosome 12 where *Bcl11b* is located exhibits frequent allelic loss in murine lymphomas [24]. *BCL11B* has been shown to play an essential role in the regulatory suppression of T-cells by regulating the expression of *FOXP3*, *IL-10* and proinflammatory cytokines [25]. *BCL11B* overexpression has been reported in an acute type of ATLL regardless of the gain/amplification of 14q32 [26]. We recently reported the expression of a *BCL11B-HELIOS* fusion gene in an ATLL patient with t(2;14)(q34;q32) [27]. These reports underscore the potential importance of *BCL11B* in T-cell maturation and in the development of T-cell malignancies. Additional information regarding its function and link to leukemogenesis is required.

We have performed a cytogenetic analysis of 50 ATLL patients and identified a chromosomal abnormality on 14q32 in 15% of the patients. In this study, a molecular analysis of one ATLL case carrying the chromosome translocations t(14;17)(q32;q22-23) was performed to identify genes that are involved in the development of ATLL. We identified *BCL11B* near the breakpoints. Notably, a dramatically decreased level of the BCL11B protein was found in many of ATLL cases and in HTLV-I-positive T-cell lines. The functional significance of *BCL11B* in leukemogenesis was demonstrated by the finding that the re-expression of *BCL11B* in ATLL-derived cell lines resulted in the arrest of their uncontrolled proliferation and cell death. These results suggest the possible involvement of *BCL11B* in the pathogenesis of ATLL.

## Materials and Methods

### Materials

This study was approved by the Institutional Review Boards of the University of Toyama and the University of Nagasaki. Written informed consent was obtained from each of the blood donors in accordance with the Helsinki Declaration. The ATLL cells were collected from the patients prior to chemotherapy treatment. The diagnosis of ATLL was based on clinical features, hematological characteristics, the presence of serum antibodies against HTLV-I antigens, and the presence of the HTLV-I proviral genome in the leukemic cell DNA. The clinical subtypes in the HTLV-I-positive group included six cases of acute leukemia, two cases of chronic leukemia. The peripheral blood mononuclear cells (PBMCs) from two healthy volunteers and eight patients with ATLL were isolated using Lympho-Prep density gradient centrifugation. Each patient exhibited more than 80% leukemic cells in the blood at the time of the analysis. The rabbit anti-human BCL11B antibody was purchased from Bethyl Laboratories Inc (Montgomery, TX). The anti-human CD4 FITC-conjugated monoclonal and the goat anti-rabbit IgG DyLight 549-conjugated secondary antibodies were purchased from Abcam (Cambridge, UK). The lentiviral packaging plasmids (pCSII-CMV, pCAG-HIVgp and pCMV-VSV-G-RSV-Rev) were provided by RIKEN BRC, which is a participant in the National Bio-Resource Project of the MEXT, Japan. The nucleotide and amino acid positions in the *BCL11B* genes were defined based on the NCBI accession number NM_138576.

### Cell Culture

Jurkat, Molt-4, PEER and SUpT-11 are HTLV-I-free human T-cell lines. HTLV-I-positive T-cell lines MT1, MT2, MT4, HUT102, ATN-1, KK1, KOB, OMT, ST1 and SO4 were examined in this study. MT-2 and MT-4 are human T-cell lines transformed *in vitro* using HTLV-I. MT-1, Hut102, ATN-1, KKI, KOB, OMT RST and SO4 are ATLL patient-derived T-cell lines. The HTLV-I-free T-cell lines were cultured in RPMI 1640 medium supplemented with 10% heat-inactivated fetal bovine serum. The HTLV-I-positive T-cell lines were cultured in RPMI 1640 medium supplemented with 10% heat-inactivated fetal bovine serum and recombinant human interleukin-2 (10 ng/ml).

### Vectors

The coding regions of the full-length human *BCL11B* gene were subcloned into the pEGFP-N1 and pCSII-CMV vectors to construct the expression vectors pCMV-BCL11B-GFP and pCSII-CMV-BCL11B-GFP, respectively. The lentiviral particles were prepared as previously described [28]. Briefly, the packaging plasmids were mixed with the pCSII-CMV-GFP or pCSII-CMV-BCL11B plasmids and were transfected using the FuGENE Transfection Reagent (Roche Diagnostics) into 293FT cells grown in a 10 cm dish. After 72 h, the supernatant containing the viral particles were harvested and concentrated by centrifugation at 28,000 rpm for 2 hours. The viral titers were determined by semi-quantitative reverse transcriptase PCR using primers to the woodchuck hepatitis virus posttranscriptional regulatory element contained within the lentiviral vectors.

### Breakpoint Analysis

Southern blotting and adaptor ligated-PCR were conducted as previously described [27]. The amplification products were cloned into the pBlueScript SK(−) plasmid vector. The clones were sequenced using the dideoxynucleotide sequencing method and an ABI3130XL DNA sequencer. The gene-specific primers used for cloning the ATLL-31 breakpoint by the adaptor ligated-PCR were 5′- CGAGAGGGGGCTTCAGGACT -3′ and 5′- CGCACCCCACCCCACCTCTT -3′. The genomic fragment containing the ATLL-31 breakpoint was amplified with primer A for *VMP1* (5′- AGGAGACGAAGCATTTGAAACTA -3′) and primer B for *BCL11B* (5′- AGGGAAAGACAGGGAGAAGA -3′).

### Mutational Analysis of BCL11B

The exons and intron/exon boundaries of the *BCL11B* were analyzed by PCR using 7 pairs of primers. The genomic DNA from each sample (25 ng) was amplified using the PrimeSTAR DNA polymerase 2XGC buffer system (Takara Bio, Japan) and the following PCR program: one cycle at 94°C for 1 min, 35 cycles at 94°C for 30 s, 65–70°C for 30 s, and 72°C for 30 s, followed by a final step at 72°C for 5 min. The primer sequences are listed in Table S1. The resulting PCR products were purified using S400 spin columns prior to being subjected to direct sequence reactions using the BigDye Terminator v3.1 Cycle Sequencing Kit (Applied Biosystems).

### Genomic Bisulfite DNA Sequencing

Genomic DNA from each sample (2 µg) was modified with sodium bisulfite using the EpiTect Bisulfate Kit (QIAGEN, Hilden, Germany) according to the manufacturer’s protocols. The modified DNA was amplified using the EpiTaq HS polymerase (Takara Bio, Japan) and the following program: one cycle at 94°C for 1 min, 30 cycles at 94°C for 30 s, 50–55°C for 30 s, and 72°C for 30 s, followed a final step at 72°C for 5 min. The PCR products were gel purified, cloned into the pBlueScript SK(−) plasmid vector and sequenced. The sequences of the oligonucleotide primers used in this study are listed in Table S2.

### Colony Formation Assay and Bromodeoxyuridine Incorporation

The ATLL-derived cell lines (OMT and KKI) and the Jurkat cells (5×10^5^ cells) were spin-infected with an equal concentration of GFP- or *BCL11B-GFP*-expressing lentivirus (moi of 25 for ATLL cells, moi of 1 for Jurkat cells) in the presence of 5 µg/ml polybrene (Sigma-Aldrich) and incubated overnight at 37°C in a 96-well plate. Five days after the infection, 1×10^5^ cells were plated on Mitomycin C treated STO feeder layers in 24-well culture plates. Seven days after the infection, equal lentiviral transduction of *BCL11B-GFP* and GFP into cells was confirmed by GFP fluorescence (Figure S1). Cells were selected with blasticidin for three weeks (Jurkat cells) or five weeks (OMT and KKI cells) and then tested for colonigenic growth. The colonies larger than 1 mm in diameter were counted. The cells were subjected to nucleofection using the Amaxa Nucleofector device set at program T-20 (Human T-Cell Nucleofector kit, Amaxa, Germany) according to the manufacturer’s protocol. Briefly, 2.5×10^6^ OMT and Jurkat cells were resuspended in 100 µl of T-cell nucleofector solution containing either 2.5 µg of pEGFP N1 control plasmid or 2.5 µg of pCMV-*BCL11B*-GFP. The OMT cells (2×10^5^) were plated on MMC-treated STO feeder layers in 24-well culture plates, and S-phase progression was monitored for 16 h at 4 days after nucleofection. The S-phase progression in Jurkat cells was monitored for 8 h at 4 days after the nucleofection. The bromodeoxyuridine (BrdU) incorporation was assayed by BrdU Labeling and Detection Kit (Roche Diagnostics).

### Immunofluorescence Analysis

The PBMCs from healthy volunteers and patients with ATLL were plated on APS-coated slides and fixed with 4% paraformaldehyde. The cells were permeabilized with PBS containing 0.1% Triton X-100 and stained with an anti-CD4 mouse monoclonal and an anti-BCL11B rabbit polyclonal antibody. The signals were visualized using a secondary antibody against mouse IgG (green) and rabbit IgG (red). The images were captured using an Olympus IX71 fluorescence microscope coupled to a SPOT RT3 digital microscopy camera (Image Solutions, UK) and were compiled using Adobe Photoshop CS5 software.

### Flow Cytometry

Fluorescence activated cell sorting (FACS) analysis was conducted on a JSAN flow cytometer (Bay Bioscience, Japan). Data were analyzed using the FlowJo software (Flow Jo, Tree Star Inc., CR, USA). Cell death was assessed by Propidium Iodide (PI) staining in the *GFP*-positive gated cell population. *GFP*-positive cell population was gated in FL1, and PI-positive cell population was analyzed in FL3. *BCL11B-GFP*- and *GFP*-positive cells were collected by FACS and subjected to mRNA extraction.

### Analysis of mRNA Expression

RNA was extracted from cells using the TRIZOL reagent (Invitrogen) according to the manufacturer’s protocol. cDNA was synthesized from 1 µg of total RNA using Superscript reverse transcriptase and an oligo(dT)_30_ primer. The semi-quantitative reverse-transcription PCR (RT-PCR) was performed at 94°C for 30 s, 60°C for 30 s, and 72°C for 30 s, after an initial denaturation step at 94°C for 1 min. Thirty cycles were performed for BCL11B and 26 cycles for 28S ribosome RNA. For the 5-aza-2′-deoxycytidine (5Az) and Trichostatin A treatments, 2×10^5^ cells were plated in 24-well culture dishes, treated with 1–5 µM of 5Az alone for 5 days and subsequently treated with or without 1 µM of Trichostatin A for an additional 24 h. The cells were harvested on day 6 after the initial 5Az treatment. The primers used for amplifying *BCL11B* were designed as follows: 5′- GGCGATGCCAGAATAGATGCCG -3′ (exon 1 sense primer) and 5′- CCAGGCCACTTGGCTCCTCTATCTCCAGAC -3′ (exon 2 antisense primer). The primers used for amplifying 28S ribosome RNA (rRNA) were designed as follows: 5′- GCCCGAAGCGTTTACTTTGA -3′ and 5′- CATGGCCTCAGTTCCGAAAAC -3′. Quantitative RT-PCR (qPCR) was performed using the *SYBR Premix Ex Taq*™ (TaKaRa) system on a 7300 *Real*-*Time PCR* System (Applied Biosystems) at 94°C for 30 s, 60°C for 30 s, and 72°C for 30 s. The results were normalized to rRNA or glyceraldehyde-3-phosphate dehydrogenase (*GAPDH*). The primers used are listed in Table S3.

### Immunoblot Analysis

The cell lysates were prepared by solubilizing cells in RIPA buffer (25 mM Tris-HCl pH 7.6, 150 mM NaCl, 1 mM Na_2_EDTA, 1 mM EGTA, 1% NP-40, 1% sodium deoxycholate). The proteins were separated by 8% SDS-PAGE and blotted onto Immobilon-P transfer membranes (Millipore, Bedford, MA). The immunoreactivity was detected using an enhanced chemiluminescence Western blotting detection system (GE Healthcare Bio-Sciences, UK), and the signals were quantified using a LAS-1000plus luminescence image analyzer and Image Gauge 3.0 software (Fujifilm, Japan).

## Results

### Mapping of the Chromosome Breakpoint

**Figure 1:**
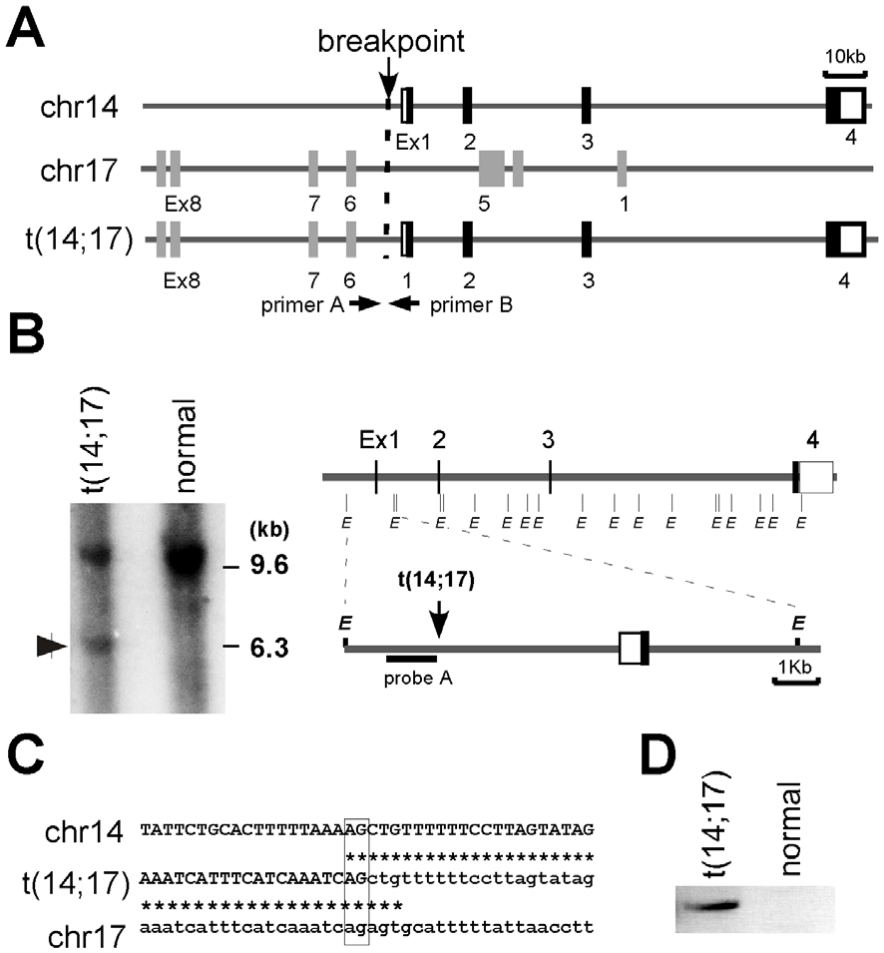
The molecular characterization of the t(14;17)(q32;q22-23) translocation in ATLL-31. (A) A schematic representation of the *BCL11B* locus on chromosome 14q32 (upper), the *VMP1* locus on chromosome 17 (middle) and the t(14;17) locus (bottom). The arrowheads indicate the primer positions used to confirm the rearrangement by PCR. (B) The Southern hybridization of *EcoR*I-digested genomic DNA from PBMCs of ATLL-31 using probe A and normal placental DNA as a control. The vertical lines denote *Eco*RI sites. (C) The nucleotide sequence analysis of the breakpoint junction exhibited in the ATLL-31 case and the alignment of the corresponding germline regions. The germline sequences of chromosomes 14 and 17 are indicated with capital and lowercase letters, respectively. The vertical lines show sequence identity. The common nucleotides at the breakpoint junction are boxed. (D) The genomic DNA from PBMCs of the ATLL-31 case and a normal volunteer was subjected to genomic PCR analysis using primers A and B to amplify the breakpoint region.

In a cytogenetic analysis of ATLL patients, we identified one case (ATLL-31) that exhibited the leukemic cell chromosomal translocations t(14;17)(q32;q22-23). These translocations were detected in more than 80% of the leukemic cells. We performed breakpoint analysis by Southern hybridization using a series of DNA probes derived from a contig of cosmid clones from the *BCL11B* locus [27]. When the Southern hybridization of the *EcoR*I-digested DNA obtained from the PBMCs of ATLL-31 was performed using probe A, which targeted the 5′ region of *BCL11B*, a unique 6.3 kb band was identified in the patient DNA but not in the normal placental DNA (Figure 1A and B). Next, we attempted to identify the breakpoint using adaptor ligated-PCR. Cloning and sequencing of the resulting PCR products revealed that the 5′ proximal region of the *BCL11B* gene was fused to intron 5 of *VMP1* in a head-to-head order with a common AG nucleotide at the breakpoint junction (Figure 1A and C). The rearrangement was further confirmed by standard PCR analysis, which was performed on undigested genomic DNA using primer A for the *BCL11B* locus and primer B for the *VMP1* locus. A specific PCR product containing the same sequence as the original adaptor ligated-PCR product was obtained from the ATLL-31 samples but not from normal PBMC samples (Figure 1D).

### Reduced BCL11B Protein Expression in ATLL Cells

**Figure 2:**
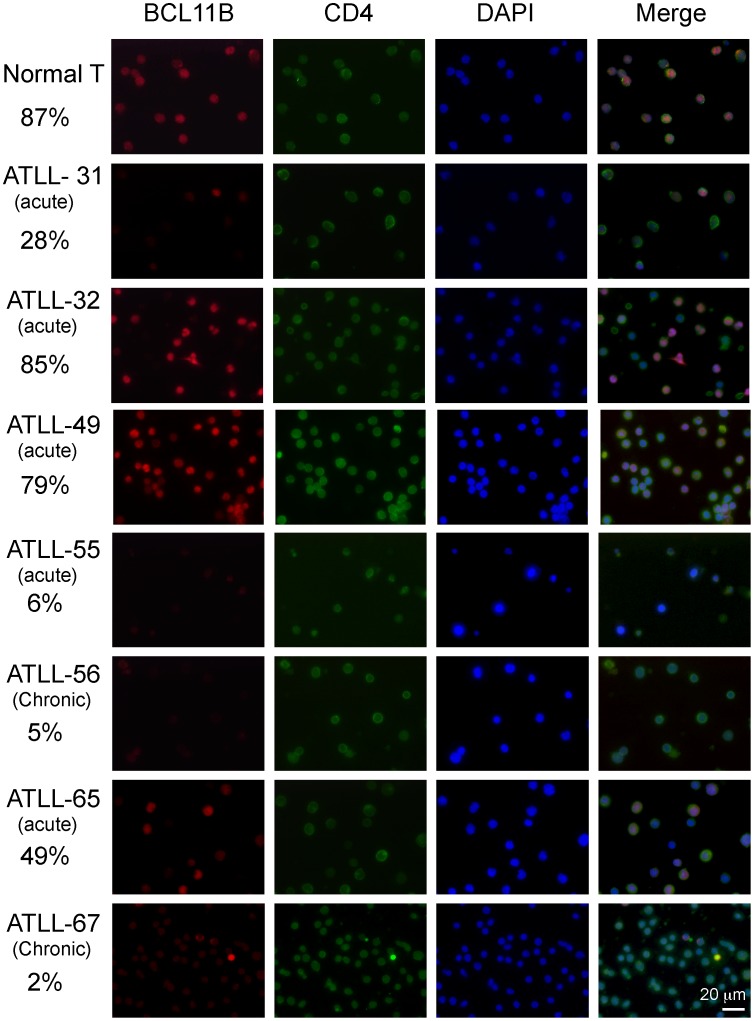
Reduced levels of BCL11B protein in ATLL patients. The immunofluorescence analysis of the BCL11B protein in PBMCs from healthy volunteer and ATLL patients are shown. The BCL11B signal was detected using a rabbit polyclonal antibody against BCL11B (red). T lymphocytes were labeled with an anti-CD4 monoclonal antibody (green). The cell nuclei were stained with DAPI. The numbers indicate the % of BCL11B-positive cells. At least one hundred cells were counted for each ATLL case.

**Figure 3:**
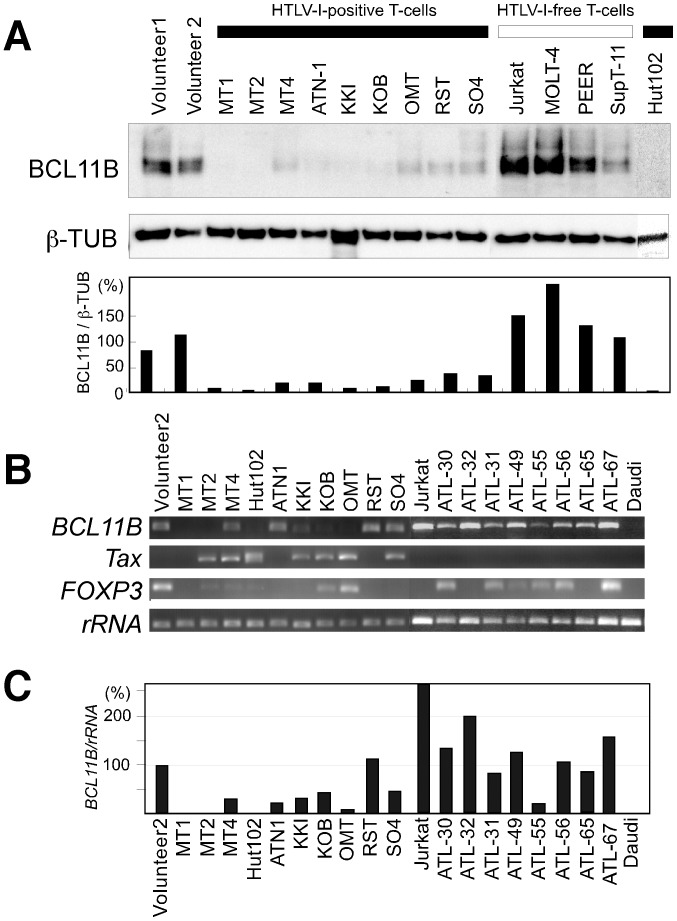
A comparative analysis of the *BCL11B* protein and mRNA expression levels. (A) Immunoblot analysis of BCL11B protein expression in a panel of HTLV-1-positive T-cell lines and HTLV-I-free T-cell lines. The cell lysates were separated by SDS-PAGE and analyzed by immunoblotting with polyclonal antibodies against BCL11B (upper panel). Jurkat, MOLT4, PEER and SupT-11 are HTLV-I-free T -lymphoid leukemia cell lines; MT-2 and MT-4 are human T-cell lines transformed *in vitro* using HTLV-I; MT-1, Hut102, ATN-1, KKI, KOB, OMT RST and SO4 are ATLL patient-derived cell lines. The data were normalized to ß-tubulin (ß-TUB) and calibrated to the BCL11B/ß-tubulin ratio in the healthy volunteer samples as a relative expression value of 100 (lower panel). (B) RT-PCR analysis of *BCL11B, Tax and FOXP3* mRNA in CD4+ T lymphocytes from healthy volunteers, HTLV-I-positive T-cell lines and HTLV-I-free T-cell lines (upper panel). (C) RT-qPCR analysis of *BCL11B* mRNA. The data were normalized to *28S* ribosome RNA (*rRNA*) and calibrated to the *BCL11B/28S*-ribosome RNA ratio in the healthy volunteer sample as a relative expression value of 100 (lower panel). The data represent the mean values of the assays, which were performed in duplicate.

To evaluate the association between the genetic abnormalities in the *BCL11B* locus and the level of BCL11B protein expression, immunofluorescence analysis was performed on uncultured leukemic cell samples from the ATLL patients (Figure 2). As a control, PBMC samples from healthy volunteers were also assessed. The double-immunofluorescence analyses revealed that 87% of CD4+ cells from healthy volunteers exhibited strong BCL11B nuclear signals with discrete punctate foci. However, in the ATLL-31 samples with t(14;17) translocation, a weak BCL11B signal was detected in only 28% of CD4+ leukemic cells, which were characterized by highly lobulated nuclei. We next examined the levels of the BCL11B protein in the other ATLL cases that did not harbor chromosomal abnormalities within the *BCL11B* locus. Strong BCL11B staining that was similar to the expression in normal T-cell was found in the ATLL-32 and ATLL-49 cases. Conversely, a very weak BCL11B signal was found in the ATLL-55, ATLL-56 and ATLL-67 cases. The ATLL-65 case was found to exhibit BCL11B-positive and BCL11B-negative cells in a 1∶1 ratio. We could not find any association with the level of BCL11B protein to clinical ATLL subtypes. We next analyzed the protein levels of BCL11B by Western blot analysis of a panel of T-cell lines (Figure 3A). Dramatically reduced levels of BCL11B protein were found in all of the HTLV-I-positive T-cell lines compared with the T-cells from healthy volunteers. Increased levels of BCL11B protein were found in the HTLV-I-free T-cell lines (Jurkat, MOLT4 PEER, and SupT-11). These results indicate that a reduction in the level of the BCL11B protein is a prevalent event in ATLL. To assess the cause underlying the downregulation of BCL11B protein in the ATLL cells, the levels of *BCL11B* mRNA were examined by RT-qPCR (Figure 3B and C). In the HTLV-I-positive T-cell lines examined, the level of *BCL11B* mRNA was categorized as either low (MT-1, MT-2, MT-4, Hut102, ATN1, KKI, KOB and OMT) or high expression (RST and SO4). Examination of the ATLL cases with weak BCL11B protein signals revealed that ATLL-55 exhibited low level of *BCL11B* mRNA expression, but ATLL-31, 56 and 67 exhibited high levels of BCL11B mRNA expression. Examination of the ATLL cases with strong BCL11B protein signals revealed that levels of *BCL11B* mRNA in the ATLL-32 and 49 were higher than that of the normal T-cells. *Tax* mRNA expression was found in many of HTLV-positive cell lines, but not in leukemic cells from patients with ATLL. We could not find any association of the mRNA level of *BCL11B* and *Tax* in these cell lines. *FOXP3* mRNA expression was found in in many of leukemic cells from patients with ATLL (6 out of 8), but hardly detectable in HTLV-I-positive T-cell lines except for KOB and OMT cells. Mutation analysis of these cases and cell lines identified no deleterious mutations in the protein coding regions or in the exon-intron junctions of *BCL11B* (Table 1). These data indicate that the *BCL11B* mRNA levels partially correlate with the protein abundance, suggesting the involvement of transcriptional and post-transcriptional control for the BCL11B protein in ATLL cells.

**Table 1:** Mutational analysis of *BCL11B* in ATLL cells.

Cell line	Promoter+Exon 1	Exon 2	Exon 3	Exon 4 (codon215–895)^2^
ATN1	None	None	None	None
OMT	Ins (CGGCGG)^1^	None	None	Silent (CCC)^3^
KKI	Del (GGC)^1^	None	None	Silent (CCC)^3^
RST	None	None	None	None
SO4	None	None	None	None
MT1	None	None	None	Silent (CCC)^3^
MT2	Ins (CGGCGG)^1^	None	None	Silent (CCC)^3^
MT4	None	None	None	None
KOB	None	None	None	None
ATLL-49	Del (GG)^1^/None	None	None	None
ATLL-55	None	None	None	Silent (CCC)^3^
ATLL-65	None	None	None	None
ATLL-67	None	None	None	None

1 −97 from start codon.

2 Codon number refers to NCBI accession number NM_138576.

3 Codon 271(Pro).

### Despite the Lack of *BCL11B* Locus Methylation in the HTLV-I-transformed T-cell Lines and in the ATL-derived Cell Lines, the Expression of *BCL11B* mRNA is Suppressed

**Figure 4:**
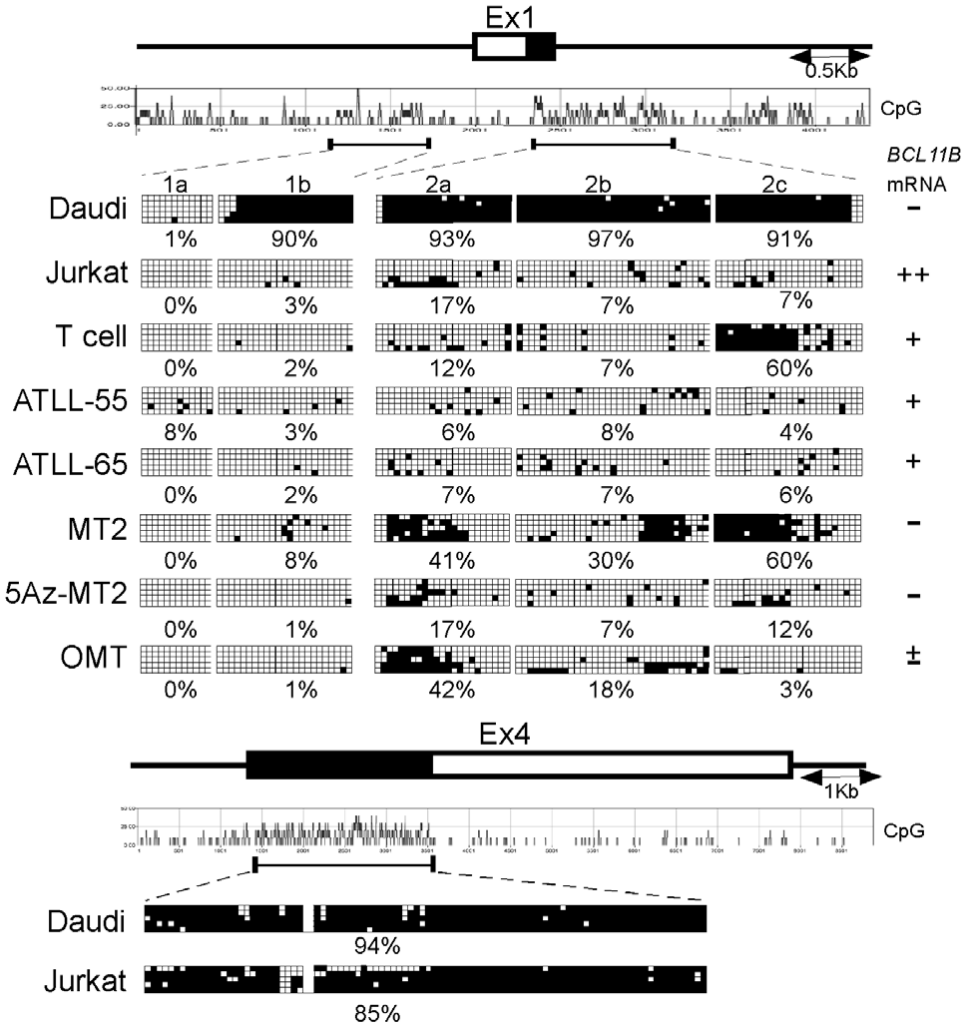
The analysis of DNA methylation of CpG islands in the *BCL11B* locus. (A) The genomic organization of the human *BCL11B* locus and the position of CpG islands. Each box indicates an individual clone sequenced in the analysis after bisulfite treatment and PCR. The open boxes indicate CpG sites at which no DNA methylation was detected. The filled boxes indicate the CpG sites at which DNA was methylated. The frequency of the CpG methylation in each region is shown. 5Az-MT2 indicates the MT2 cells treated with 5Az.

We found that *BCL11B* mRNA expression is repressed in many of HTLV-I-positive T-cell lines and leukemic cells from ATLL-55. We next dissected the molecular mechanism underlying the epigenetic silencing of *BCL11B*. The *BCL11B* locus harbors three CpG islands, including the 5′ upstream region (1a and 1b), exon 1 and intron 1 (2a, 2b and 2c) and the protein-coding region of exon 4. To examine whether the *BCL11B* gene methylation status in these regions correlates with *BCL11B* mRNA expression levels, we performed bisulfite mapping of the CpG islands (Figure 4). This analysis revealed that the 5′ upstream region (0% in 1a and 3% in 1b) and the 1st intron (17%, 7% and 7% in 2a, b and c) were demethylated in the Jurkat cells, whereas in contrast, dense methylation in 1b and 2a-c (90%, 93%, 97% and 91%) but not in 1a (1%) was apparent in the Daudi cells. The CpG sites of exon 4 were hypermethylated in the Jurkat (85%) and Daudi cells (94%), suggesting that regions 1b and 2a-c are primary candidate regions responsible for the regulation of *BCL11B* gene expression. Normal T lymphocytes were found to be unmethylated in 1a, 1b, 2a and 2b (0%, 2%, 12% and 7%) but exhibited relatively high methylation in 2c (60%). The methylation status of ATLL-55 with low levels of *BCL11B* mRNA expression was similar to that observed in the Jurkat cells. Relatively increased methylation (40%) was found in 2a in two cell lines that did not express *BCL11B* mRNA (MT2 and OMT), suggesting the possible involvement of this region in the suppression of *BCL11B* mRNA expression. Although the treatment of MT2 cells with 5Az reduced methylation at 2a, b and c, the 5Az treatment alone or in combination with Trichostatin A failed to increase the levels of *BCL11B* mRNA (Figure S2). These results suggest that methylation in these regions likely does not play a role in the epigenetic regulation of *BCL11B* in the MT-2 cells.

### The Expression of *BCL11B* Results in the Growth Suppression of Cell Lines Established from ATLL Patients

**Figure 5:**
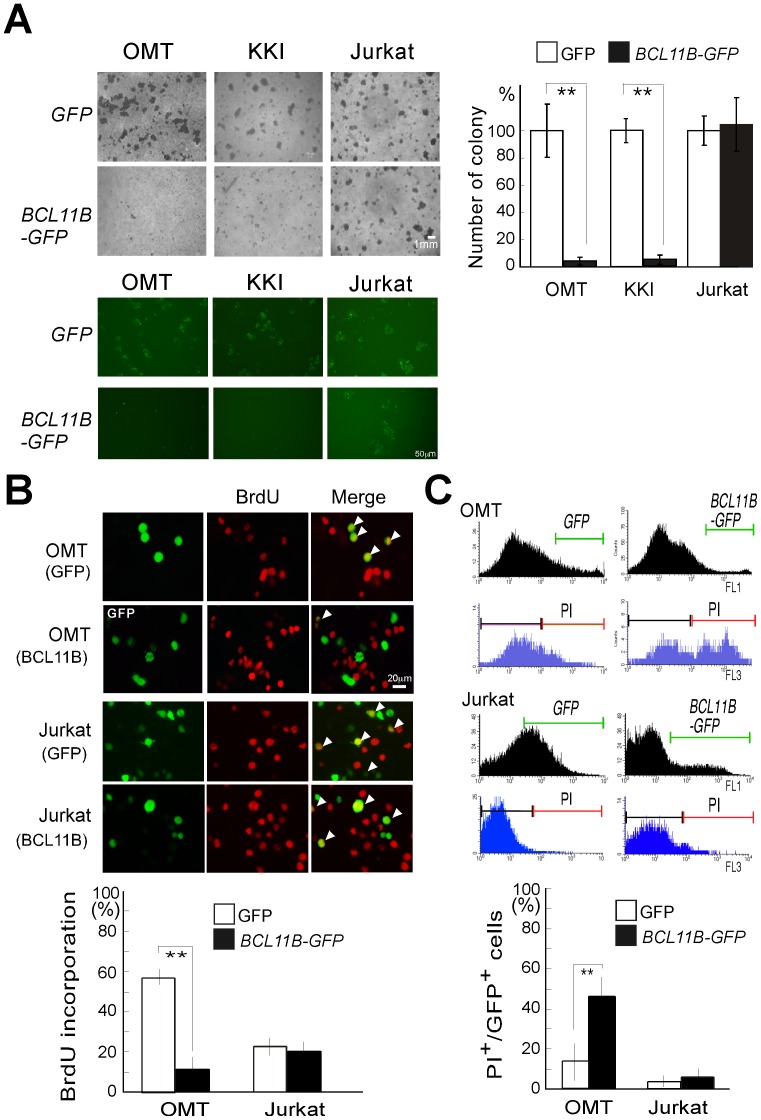
The expression of BCL11B suppresses the growth of ATLL-derived cells. (A) The growth inhibitory effects of *BCL11B* expression in the ATLL-derived T-cell lines as determined by a colony survival assay. The Jurkat cells and the ATLL-derived cell lines (OMT and KKI) were lentivirally transduced with equal concentrations of a *BCL11B-GFP* or *GFP* virus and selected with blasticidin for 2–5 weeks. Representative images of the drug-resistant colonies in Jurkat, OMT and KKI cells are shown (left upper panel). The ectopically expressed GFP or *BCL11B-GFP* signal after the blasticidin selection was confirmed by fluorescent microscope (left lower panel). The relative number of colonies in the cells transduced with *BCL11B* was determined as the percentage of those transduced with the control GFP virus. (n = 3, **p<0.01 by Student’s t-test) (right upper panel). (B) The expression of *BCL11B* led to the suppression of DNA synthesis in OMT cells but not in Jurkat cells. The OMT and Jurkat cells were nucleofected with *GFP* or *BCL11B-GFP* expression plasmids. Cells entering the S phase were detected using an anti-BrdU mouse monoclonal antibody (red). The GFP or BCL11B-GFP signals were detected with an anti-GFP antibody (green). The arrows indicate colocalization. The graph indicates the percentage of nuclei that incorporated BrdU in BCL11B-GFP- or GFP-positive cells. At least 100 BCL11B-GFP- or GFP-positive cells were scored for each sample in three independent experiments, and the frequency of the cells in S phase was determined by the quantitation of the BrdU-positive nuclei. (n = 3, **p<0.01 by Student’s t-test). (C) Cell death assessed by PI staining in the *BCL11B*-GFP-expressing cells**.** The OMT and the Jurkat cells were stained with PI at 4 days after the nucleofection and the percentage of dead cells (PI-positive) in BCL11B-GFP- or GFP-positive cells was determined by FACS. At least 5000 BCL11B-GFP- or GFP-positive cells were scored for each sample in three independent experiments, and the frequency of PI^+^ cells in *BCL11B-GFP* ^+^ or GFP^+^ cells was determined (n = 3, **p<0.01 by Student’s t-test).

To establish a more causal relationship between *BCL11B* and leukemogenesis, we analyzed whether *BCL11B* expression might block cellular proliferation in ATLL-derived cell lines. Gene expression was achieved through the lentiviral transduction of either *BCL11B-GFP* or *GFP* into ATLL-derived cell lines (OMT and KKI) and Jurkat cells, after which the cell growth was measured using colony formation assays. Although BCL11B-GFP or GFP signals were found in OMT and KKI cells at seven days after the transduction, only GFP-expressing cells formed colonies after drug selection (Figure S1 and Figure 5A). No statistically significant difference in growth was observed between *BCL11B-GFP-* and *GFP*-transfected Jurkat cells. We assessed the percentage of cells undergoing *de novo* DNA synthesis using a BrdU incorporation experiment in the *BCL11B-GFP* or *GFP*-nucleofected OMT and Jurkat cells. As shown in Figure 5B, the expression of *BCL11B-GFP* in OMT cells was associated with a strong decrease in BrdU incorporation compared with the *GFP* control cells. *BCL11B-GFP* expression in Jurkat cell did not show significant effect on BrdU incorporation compared with the *GFP* control cells. We next analyzed whether *BCL11B* expression might influence cell viability. As shown in Fig. 5C, an increase in PI-positive cells was clearly increased in *BCL11B-GFP-* expressing OMT cells compared to that of control *GFP*-expressing OMT cells. No statistically significant difference in cell viability was observed between *BCL11B-GFP-* and *GFP*-expressing Jurkat cells (Fig. 5C). These results suggest that *BCL11B* expression can significantly suppress the growth of ATLL cell lines but does not suppress that of Jurkat cells. We next analyzed whether the *BCL11B* could modulate the expression of known *BCL11B* target genes, as well as the expression of cell cycle regulatory- and inflammatory-related genes. Notably, the most significantly induced gene was *FOS*, the transcription of which is primed by growth stimuli and genotoxic stress. The overexpression of *BCL11B-GFP* elicited marginal effects on the transcription of G1-S phase-specific genes. Among known *BCL11B* target genes examined, IL2 mRNA was up-regulated in the *BCL11B*-overexpressing cells (Table 2).

**Table 2:** *BCL11B* expression induces *FOS* expression in the OMT cells.

Gene Symbol	Gene Title	Fold change
		(mean±S.D)
*FOS*	FBJ murine osteosarcoma viral oncogene homolog	8.3±5.3
*FOSB*	FBJ murine osteosarcoma viral oncogene homolog B	1.5±0.4
*FRA1*	FOS-like antigen 1	1.3±0.4
*FRA2*	FOS-like antigen 2	1.2±0.3
*JUN*	jun proto-oncogene	2.0±0.3
*JUNB*	jun B proto-oncogene	1.0±0.3
*JUND*	jun D proto-oncogene	1.0±0.4
*BCL6*	B-cell CLL/lymphoma 6	1.7±0.1
*GATA3*	GATA binding protein 3	1.0±0.2
*TCF8*	zinc finger E-box binding homeobox 1	2.0±0.4
*IKAROS*	IKAROS family zinc finger 1	1.2±0.2
*HELIOS*	IKAROS family zinc finger 2	1.6±0.4
*FOXP3*	forkhead box P3	0.7±0.2
*PCNA*	proliferating cell nuclear antigen	1.1±0.4
*CCND1*	cyclin D2	0.9±0.3
*CCND3*	cyclin D3	1.2±0.2
*CCNE1*	cyclin E1	0.9±0.3
*CCNA2*	cyclin A2	0.9±0.2
*CDK2*	cyclin-dependent kinase 2	1.0±0.4
*CDK4*	cyclin-dependent kinase 4	1.0±0.4
*E2F1*	E2F transcription factor 1	1.0±0.5
*E2F2*	E2F transcription factor 2	0.9±0.5
*CDKN1A*	cyclin-dependent kinase inhibitor 1A (p21, Cip1)	1.1±0.2
*CDKN1B*	cyclin-dependent kinase inhibitor 1B (p27, Kip1)	1.0±0.2
*CDKN1C*	cyclin-dependent kinase inhibitor 1C (p57, Kip2)	ND
*TP53*	tumor protein p53	1.3±0.4
*IL2*	interleukin 2	2.6±1.6
*CCR4*	chemokine (C-C motif) receptor 4	0.9±0.3
*IL10*	interleukin 10	ND
*ICAM* *PTP1C*	intercellular adhesion molecule 1protein tyrosine phosphatase, non-receptor type 6	0.9±0.20.8±0.2
*MYB*	v-myb myeloblastosis viral oncogene homolog (avian)	1.1±0.5
*Tax*	HTLV-1 regulatory and accessory genes	1.2±0.4
*HBZ*	HTLV-1 bZIP factor gene	1.0±0.3

The OMT cells were transfected with plasmids encoding *GFP* (control) or *BCL11B-GFP*. The cells with GFP fluorescence were isolated by FACS at 48 h after the transfection, and total RNA was extracted. The mRNA levels were quantified by RT-PCR, normalized to GAPDH and expressed relative to the GFP-expressing control cells.

## Discussion

By characterizing the 14q32 breakpoint of one ATLL case, we identified the *BCL11B* gene, which resides near the breakpoint. We demonstrated that the level of the BCL11B protein in ATLL cells was reduced by several mechanisms of transcriptional and post-transcriptional regulation. The ectopic expression of the *BCL11B* gene revealed its growth suppressor activity in ATLL-derived cell lines but not in Jurkat cells. Thus, our findings lead us to propose that the reduction of BCL11B protein levels is associated with the development of ATLL.

We found that *BCL11B* mRNA expression is repressed in many of HTLV-I-positive T-cell lines and leukemic cells from ATLL-55. Our bisulfate study revealed that the CpG islands in the promoter, exon 1 and intron 1 of *BCL11B* are unmethylated in *BCL11B*-expressing and non-expressing T-cell lines. Given that methylation-free CpG islands are usually associated with open chromatin structures, this result raises the question of how *BCL11B* mRNA expression is repressed in the HTLV-I-related cell lines (MT2 and OMT) and leukemic cells from ATLL-55. One possible explanation is that the unmethylated status of the CpG islands might be permissive for transcription. The levels of *BCL11B* mRNA expression from the unmethylated *BCL11B* locus might depend on the degree of its association with particular transcription factors.

A relatively increased level of *BCL11B* mRNA expression was previously reported in acute-type ATLL cases [26]. However, the immunofluorescent and Western blot analysis revealed that BCL11B protein levels partially reflect the *BCL11B* mRNA levels in the ATLL cases and HTLV-I-positive T-cell lines. The mechanisms underlying this discrepancy remain unknown. But considering the low frequency of *BCL11B* abnormalities in the ATLL cases and the HTLV-I-positive T-cell lines, translational modifications might be responsible for the downregulation of the BCL11B protein. *BCL11B* has a long 3′ untranslated region, which might serve to regulate the translation through the binding of microRNAs or RNA-binding proteins. Recently, the deregulation of microRNAs including miR-93 and let-7 has been reported in ATLL cells [7], [29], [30]. As BCL11B mRNA transcripts contain target sites for miR-93 and let-7, these microRNAs might contribute to the downregulation of the BCL11B protein in ATLL cells.

The role of *BCL11B* in the pathogenesis of hematological diseases is controversial. *BCL11B* has been described to elicit an oncogenic function in a subset of tumors. Increased levels of *BCL11B* expression have been linked to human T-cell acute lymphoblastic leukemia, and the inhibition of *BCL11B* expression results in the apoptosis of malignant T-cells [26], [31]. The induction of *BCL11B* expression correlates with reduced differentiation status in head and neck squamous cell carcinomas [32]. Furthermore, *BCL11B*-knock down Jurkat cells induce apoptosis, whereas *BCL11B*-overexpressed Jurkat cells induce a cell-cycle arrest under genotoxic stress, consistent with the oncogenic properties of *BCL11B* [33], [34]. On the other hand, the haploinsufficient tumor suppressor function of the *BCL11B* gene has been reported in mouse and human lymphomalignancies [20], [24], [35], [36]. The ectopic expression of *BCL11B* in BCL11B-negative HeLa and hematopoietic progenitor FDC-P1cell lines suppress tumor growth by inducing cell cycle arrest [37]. These data support the tumor suppressor function of *BCL11B* [24]. Although the biological function of *BCL11B* is still a matter of debate, *BCL11B* might regulate cell-cycle progression through DNA damage response depending on the cellular context and the expression level [34], [38]. Because transformed HTLV-I-free T-cells express abundant BCL11B protein, these cells might utilize this protein to induce transient cell-cycle arrest, thereby allowing time for DNA repair and cell survival under DNA stress condition. Contrary, the frequent suppression of BCL11B protein expression was found in most of the HTLV-I-positive cells regardless of *Tax* expression, implying that the low level of BCL11B protein is advantageous for survival and growth-promotion in these cells. We found that the significant growth suppression and cell death effects elicited by *BCL11B* expression in two ATLL-related cell lines. These results are in good agreement with this idea. ATLL cells exhibit severe chromosomal aberrations and often exhibit anti-apoptotic phenotypes. These phenotypes can be maintained by inactivating apoptotic death pathways and cell-cycle checkpoints that are normally engaged by the DNA damage response [5]. The mechanisms by which *BCL11B* execute growth suppression in ATLL-related cells is still unknown. But given our observation that *FOS*, but not other genes involved in the G1-S transition, was significantly induced by the expression of *BCL11B*, we speculate that ectopically expressed *BCL11B* can potentiate the DNA damage responses that are normally suppressed in HTLV-I-positive T-cells and induce persistent cell-cycle arrest leading to cell death [39], [40]. Alternatively, given that some ATLL cases express abundant BCL11B protein, *BCL11B* might be involved in only some aspects of ATLL development. In such cases, mechanisms other than the down-regulation of BCL11B protein might be predominant for leukemogenesis.

## Supporting Information

Figure S1 Lentiviral transduction efficiency in OMT, KKI and Jurkat cells. Lentiviral transduction of *BCL11B-GFP* or GFP into cells was confirmed by fluorescent microscope seven days after the infection.
(TIF)

Figure S2 The TSA and/or 5′-Aza treatments failed to increase the *BCL11B* mRNA levels. The cells were treated with 5Az alone or in combination with Trichostatin A and subjected to semi-quantitative RT-PCR analysis for the expression of *BCL11B*. *GAPDH* served as a loading control.
(TIF)

Table S1 Primers used in the mutation analysis. The exons and intron/exon boundaries of the *BCL11B* were amplified from genomic DNA using PrimeSTAR DNA polymerase 2XGC buffer system with pairs of gene-specific primers.
(DOCX)

Table S2 Primers used in the bisulfate PCR. Genomic DNA modified with sodium bisulfite was amplified using the EpiTaq HS polymerase with pairs of bisulfite-specific primers designed for the amplification of the bisulfite-converted DNA.
(DOCX)

Table S3 Primers used in the RT-PCR. Known *BCL11B* target genes, as well as cycle regulatory- and inflammatory-related genes were amplified by RT-PCR with pairs of gene-specific primers.
(DOCX)
