# Supplementary figures and images for: Reduced Level of the BCL11B Protein Is Associated with Adult T-Cell Leukemia/Lymphoma
Source: PLoS One. 2013 Jan 30;8(1):e55147. doi: 10.1371/journal.pone.0055147 (PMC3559337; doi:10.1371/journal.pone.0055147)

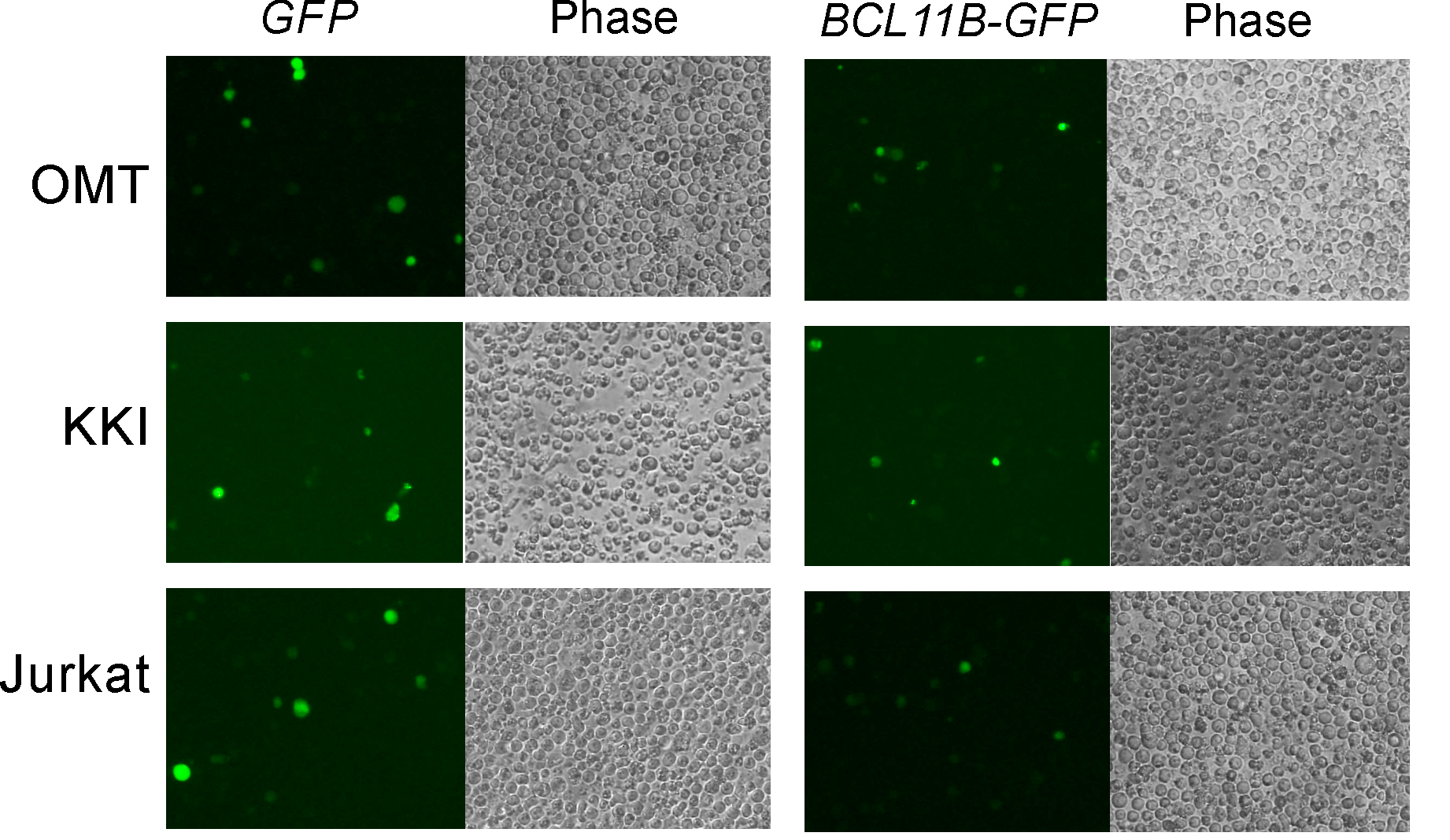

Supplement: Figure S1 — Lentiviral transduction efficiency in OMT, KKI and Jurkat cells. Lentiviral transduction of BCL11B-GFP or GFP into cells was confirmed by fluorescent microscope seven days after the infection. (TIF) [file pone.0055147.s001.tif]

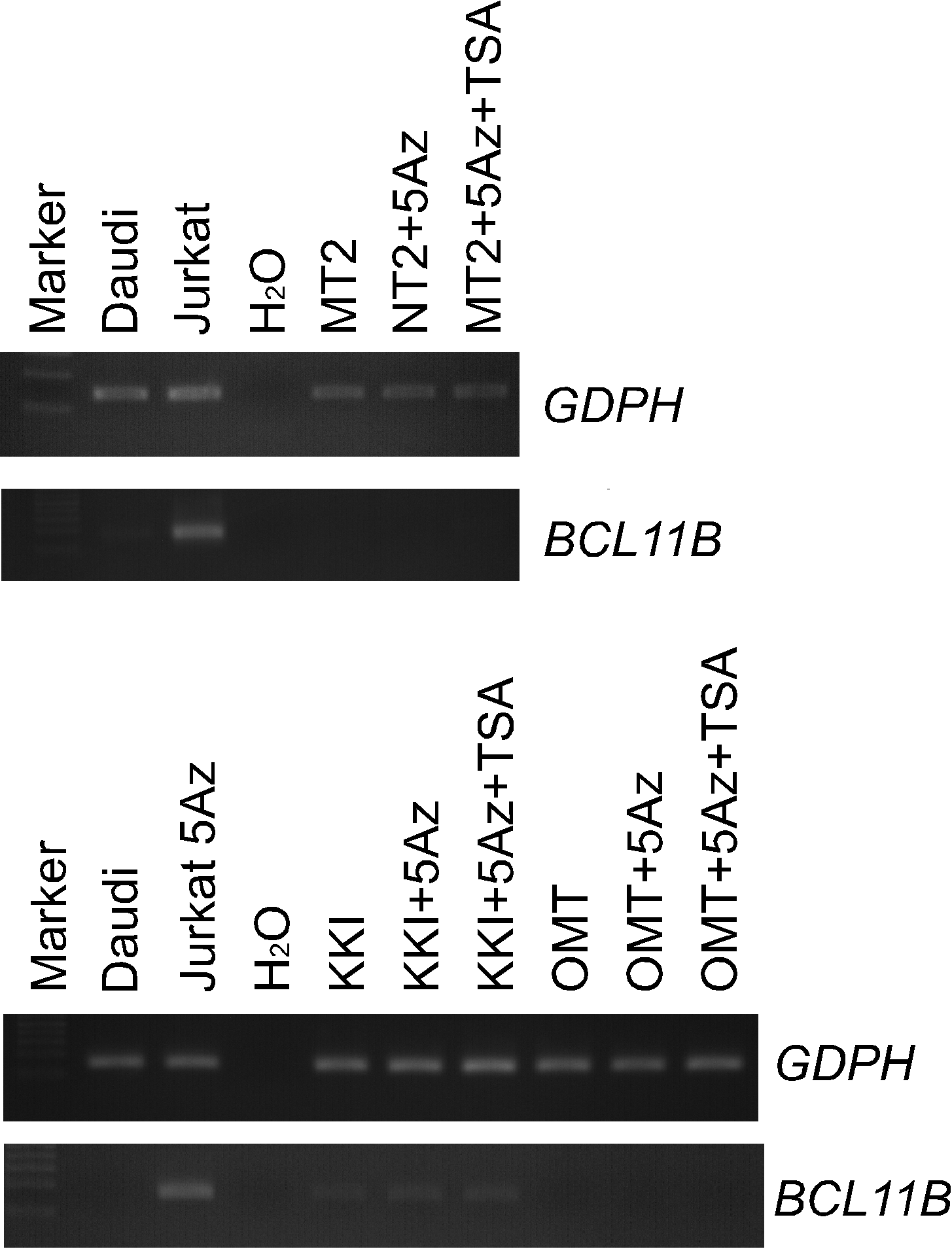

Supplement: Figure S2 — The TSA and/or 5′-Aza treatments failed to increase the BCL11B mRNA levels. The cells were treated with 5Az alone or in combination with Trichostatin A and subjected to semi-quantitative RT-PCR analysis for the expression of BCL11B. GAPDH served as a loading control. (TIF) [file pone.0055147.s002.tif]
