# Supplementary material for: Reduced Level of the BCL11B Protein Is Associated with Adult T-Cell Leukemia/Lymphoma
Source: PLoS One. 2013 Jan 30;8(1):e55147. doi: 10.1371/journal.pone.0055147 (PMC3559337; doi:10.1371/journal.pone.0055147)
Supplement: Table S1 — Primers used in the mutation analysis. The exons and intron/exon boundaries of the BCL11B were amplified from genomic DNA using PrimeSTAR DNA polymerase 2XGC buffer system with pairs of gene-specific primers. (DOCX) [file pone.0055147.s003.docx]

**Table S1. Primers used in the mutation analysis**

| PCR sense primer | | PCR antisense primer | |
| --- | --- | --- | --- |
| Ex1U | AGACATTTGCAAGTGCAAGAGGCCAAGCAG | Ex1L | CAGGCCGACGCCGTAGACTCTGCCAGC |
| Ex2U | GAAGGGGTGGACCGGAAGTTGGGTGCTGTG | Ex2L | TCCTGAAGGTCACCTGGATGGTGGACCCTC |
| Ex3U | AGCCCAGCCTGGCAGCTGTGGAAACCG | Ex3L | CTTCCTCCCTGGGTCCCCACCTTGCTC |
| Ex4 1U | CTGCAGCCCCTGTCAGGCAGGGCAC | Ex4-1L | GTTCAGGAGCCGGTGCATAGGGTT |
| Ex4 2U | CTTCGACCGAGTCATGCGCCTGAA | Ex4-2L | CTTCTGCTTGTCGGCCAGGAGCTC |
| Ex4 3U | AGCTGCTACTGGAGAACGAGAGCC | Ex4-3L | GTTCTCGGACGAGTGCTCGGACGA |
| Ex4 4U | AAGGTGGAGAAGGACCTGGAGCTG | Ex4-4L | GCAGACACAGGTTAGGTTGGAGTG |
