# Supplementary material for: Reduced Level of the BCL11B Protein Is Associated with Adult T-Cell Leukemia/Lymphoma
Source: PLoS One. 2013 Jan 30;8(1):e55147. doi: 10.1371/journal.pone.0055147 (PMC3559337; doi:10.1371/journal.pone.0055147)
Supplement: Table S2 — Primers used in the bisulfate PCR. Genomic DNA modified with sodium bisulfite was amplified using the EpiTaq HS polymerase with pairs of bisulfite-specific primers designed for the amplification of the bisulfite-converted DNA. (DOCX) [file pone.0055147.s004.docx]

**Table S2. Primers used in the bisulfate PCR**

| Bisulfate PCR sense primer | | Bisulfate PCR antisense primer | |
| --- | --- | --- | --- |
| CpG 1aS | TAGAGGTAGTTTTAGTTATAGAGAGAT | CpG 1aAS | ACTCCTTCCCAATTCACCTAACAAAC |
| CpG 1bS | TTGTGTGTGTGTGTGTGTGAGTGTG | CpG 1bAS | ATCTCTCTATAACTAAAACTACCTCTA |
| CpG 2aS | AGGGAAAAAAAATATTAATTTGGGTAGAGG | CpG 2aAS | ATAATAAACTCCCTCTAAAACAAATACTAC |
| CpG 2bS | GGTAATTTGTAGTATTTGTTTTAGAGGGAG | CpG 2bAS | CACACACACACACTCCTCCAACCTACATAC |
| CpG 2cS | AGTTGGGAAGTGGGGAAAAGTTGG | CpG 2cAS | AAAAAACAACTACAAAATCCTAAAACTAAC |
| CpG Ex4 1S | TGTTTGTAGGTTAGTAAGTTTAAG | CpG Ex4 1AS | ACTCTCATTCTCCAATAACAACTCC |
| CpG Ex4 2S | GAGGAGGAGGAGTTGTTATTGGAGA | CpG Ex4 2AS | TTCTCCATAACCTTACCCAACACC |
| CpG Ex4 3S | TTGGTGTTGGGTAAGGTTATGGA | CpG Ex4 3AS | ACAACTCCAAATCCTTCTCCACCT |
