# Supplementary material for: Reduced Level of the BCL11B Protein Is Associated with Adult T-Cell Leukemia/Lymphoma
Source: PLoS One. 2013 Jan 30;8(1):e55147. doi: 10.1371/journal.pone.0055147 (PMC3559337; doi:10.1371/journal.pone.0055147)
Supplement: Table S3 — Primers used in the RT-PCR. Known BCL11B target genes, as well as cycle regulatory- and inflammatory-related genes were amplified by RT-PCR with pairs of gene-specific primers. (DOCX) [file pone.0055147.s005.docx]

**Table S3. Primers used in the RT-PCR**

| Gene | Sense primer | Antisense primer |
| --- | --- | --- |
| *FOS* | TACTACCACTCACCCGCAGACTC | CTTTTCCCTTCGGATTCTCCTTTT |
| *FOSB* | TAGCAGCAGCTAAATGCAGGAAC | CCAGCTGAAGCCATCTTCCTTAG |
| *FRA1* | CAGTGGATGGTACAGCCTCATTT | GCCCAGATTTCTCATCTTCCAGT |
| *FRA2* | CCAGCAGAAATTCCGGGTAGATA | TCTCCTCCTCTTCAGGAGACAGC |
| *JUN* | GCAAACCTCAGCAACTTCAACC | TGTCTGAGGCTCCTCCTTCAGG |
| *JUNB* | AAAATGGAACAGCCCTTCTACCA | AGCCCTGACCAGAAAAGTAGCTG |
| *JUND* | AACACCCTTCTACGGCGATGAG | GGGTAGAGGAACTGTGAGCTCGT |
| *BCL6* | GCCCATGTGC TTATCCACAC | CCATGCTTCA GCAGGCTTTG |
| *GATA3* | AAGGCATCCAGACCAGAAACCG | AGCATCGAGCAGGGCTCTAACC |
| *TCF8* | ACCTTTGCATACAGAACCCAAC | TCTTGCAGTTTGGGCATTCA |
| *IKAROS* | TCTTCGCCCC CGAGGATCA | TGCAGAGGTGGCATTTGAAG |
| *HELIOS* | TTATCTTCAGCCCGACATT | TTGGGTGTGCTTGAGGTGAG |
| *FOXP3* | CGACCCCCTTTCACCTACGCCACGCTCATC | CACAGCCCCCTTCTCGCTCTCCACCCGCAC |
| *PCNA* | AGGGCTCCATCCTCAAGAAGG | TGGTGCTTCAAATACTAGCGC |
| *CCND1* | GCTCCTGGTGAACAAGCTCAA | TGGCACAAGAGGCAACGA |
| *CCND2* | TGGAGCTGCTGTGCCACG | GTGGCCACCATTCTGCGC |
| *CCND3* | AGGCCTGGGTGCCTGCTCCAT | CTACAGGTGTATGGCTGTGAC |
| *CCNE1* | CAAGTACACCAGCCACCTC | GTACAACGGAGCCCAGAA |
| *CCNA2* | AGCTGCCTTTCATTTAGCACTCTAC | TTAAGACTTTCCAGGGTATATCCAGTC |
| *CCNB1* | TCTGGATAATGGTGAATGGACA | CGATGTGGCATACTTGTTCTTG |
| *E2F1* | CACAGATCCCAGCCAGTCTCTA | GAGAAGTCCTCCCGCACATG |
| *E2F2* | AAGAAGTTCATTTACCTCCTGA | AATCACTGTCTGCTCCTTAAA |
| *E2F3* | TGGTACCATTGAGTTGCTGCTATT | AGCTCATGTGTTGCCCTTTATACA |
| *CDKN1A* | TGGAGACTCTCAGGGTCGAAA | GGCGTTTGGAGTGGTAGAAATC |
| *CDKN1B* | CGCCATATTGGGCCACTAA | CGCAGAGCCGTGAGCAA |
| *CDKN1C* | TCACCGCAGCCTCTTGCGC | GGATTTCGGGACGTCCCGC |
| *TP53* | GTTCCGAGAGCTGAATGAGG | TCTGAGTCAGGCCCTTCTGT |
| *IL2* | CAACTGGAGCATTTACTGCTGGA | TCAGTTCTGTGGCCTTCTTGG |
| *CCR4* | AAGAAGAACAAGGCGGTGAAGATG | AGGCCCCTGCAGGTTTTGAAG |
| *IL10* | AATAAGGTTTCTCAAGGGGCT | AGAACCAAGACCCAGACATCAA |
| *ICAM1* | CCGGAAGGTGTATGAACTG | TCCATGGTGATCTCTCCTC |
| *PTP1C* | GGAGTCGGAGTACGGGAACAT | ATCCTCCTTGTGTTTGGACGA |
| *MYB* | CAGTGACGAGGATGATGAGGACT | AACGTTTCGGACCGTATTTCTGT |
| Tax | CCGGCGCTGCTCTCATCCCGGT | GGCCGAACATAGTCCCCCAGAG |
| HBZ | GAATTGGTGGACGGGCTATTATC | TAGCACTATGCTGTTTCGCCTTC |
| GAPDH | GAAGGTGAAGGTCGGAGTC | GAAGATGGTGATGGGATTTC |
